# Enhancing the Chain of Survival: The Role of Smartphone Applications in Cardiopulmonary Resuscitation

**DOI:** 10.7759/cureus.68600

**Published:** 2024-09-04

**Authors:** Lydia Vallianatou, Theodoros Kapadohos, Maria Polikandrioti, Evangelia Sigala, Evangelia Stamatopoulou, Eleni-Marina Kostaki, Pavlos Stamos, Dimitra Koutsavli, Antonia Kalogianni

**Affiliations:** 1 Research, University of West Attica, Athens, GRC; 2 Department of Nursing, University of West Attica, Athens, GRC; 3 Department of Nursing, Postgraduate Program, Applied Clinical Nursing, University of West Attica, Athens, GRC; 4 Nursing Education Office, Evangelismos General Hospital, Athens, GRC; 5 Catheterization Laboratory, University Hospital of Athens Attikon, Athens, GRC; 6 Department of Occupational Therapy, University of West Attica, Athens, GRC; 7 Informatics, Hellenic American University, Athens, GRC

**Keywords:** basic life support (bls), public health, bystander cpr training, smartphone applications, real-time cpr guidance

## Abstract

This review explores the role of smartphone applications in providing real-time guidance for cardiopulmonary resuscitation (CPR) practices and highlights their potential to improve CPR quality among laypersons. A narrative literature review was conducted on the effectiveness of mobile CPR applications for smartphones. Studies published between 2014 and 2024 were included to ensure that new technological advances were examined. Our findings revealed that guided CPR applications significantly improve most critical parameters for efficacious resuscitation. Application users demonstrated that they achieved performance comparable to or even better than CPR-certified individuals. However, these tools have limitations, mostly related to familiarity, which may result in a delay in activating the application and, therefore, in initiating CPR.

While smartphone applications are promising tools for enhancing bystander CPR, their integration into emergency medical response requires careful consideration. To fully take advantage of these applications, they should be incorporated into public health campaigns and standard CPR training. This would be even more successful if the application's functionality were standardized across different regions. Our research indicates that a combination of education and technology will likely play a major role in CPR training in the future, improving the efficacy and accessibility of life-saving measures. Smartphone applications could greatly improve the chain of survival in out-of-hospital cardiac arrest (OHCA) events. The design and accessibility of these applications as well as the integration of these applications with current emergency response frameworks should be the main areas of future research.

## Introduction and background

Out-of-hospital cardiac arrest (OHCA) is a global health concern [1,2]. In Europe, cardiac arrest is the third leading cause of death [3]. In 2017, 37,054 cases of OHCA were recorded in Europe in a three-month recording of the EuReCa TWO study [4]. In 2022, the CARES study reported 147,736 OHCAs in the United States, covering 53% of the total population, representing approximately 178 billion people [5]. Also in the USA, primary mortality from sudden cardiac death (SCD) was 19,427 out of 43,6852 reported SCD cases in 2020 [6]. The risk of SCD increases with age (1-10 years of age: 0.49 per 100.000 individuals vs. 26-34 years of age: 2.76 per 100.000 individuals) [6]. According to reversible rhythms of cardiac arrest, the frequency of ventricular fibrillation (VF) arrests is 15,09-20,16 per 100,000 person-years, whereas the frequency of all-rhythm cardiac arrests [VF, ventricular tachycardia (VT), asystole, pulseless electrical activity (PEA)] treated by emergency medical services (EMS) is 36.77-46,17 per 100,000 person-years.

In a US study, the majority of young athletes who suffered sudden cardiac arrest (SCA) during competitive sports had primary rhythm VF and VT. In this study, 43.8% were discharged from the hospital [6,7]. In general, the survival rates for cardiac arrests with VF are 21.2%, and they are 10.7% for all-rhythm cardiac arrests [8]. In cases of early identification, immediate CPR, and defibrillation in three to five minutes, survival with good neurological outcome rates increases significantly to 60% or more [9-11]. OHCA is confirmed in a collapsed victim who ceases to breathe for 10 seconds, after checking by opening the airway [9]. Most cases will occur at home (73%) or in public areas (16%), with a mortality rate twice as high compared to in-hospital cardiac arrests (IHCAs, 18% vs. 9%) [6].

A study in 28 European countries found that when bystanders [bystander CPR (BCPR), the person who is present at the scene and initiates CPR] or non-medical persons initiate CPR, the proportion of victims who recover spontaneous circulation increases compared to when CPR is initiated by trained health system rescuers (when they arrive at the scene) [12]. This shows that the speed of initiation of CPR is a critical aspect of the rescue process [12]. However, in most countries, the BCPR rate is less than 10% and the probability of survival decreases by 7-10% for every minute of delayed action [3,12]. Hence, the European Resuscitation Council (ERC) [9], the American Heart Association (AHA) [13], and the International Liaison Committee on Resuscitation (ILCOR) [14] have emphasized providing hands-only CPR as BCPR, if they feel unqualified to provide rescue breaths (or if their insecurity about giving rescue breaths causes delays). Community awareness campaigns are essential for advancing this understanding [15].

Technology has great made strides in the healthcare industry, and many applications in the area of citizen emergency response have been introduced [16]. Digital tools constitute an ideal solution for providing immediate guidance to users, personalized information for chest compression performance, providing the location of automated external defibrillators (AEDs), or calling EMS [17,18]. Social media, mobile devices, and smart wearables provide creative means for the general public to recognize and intervene earlier in cases of SCAs, both when they are seen and when they are not, boosting the rate of effective CPR. Many applications are available for free download [1]. PocketCPR, for instance, demonstrates adult CPR to a bystander and instructs them to hold their smartphone in their hand to ensure they are applying compressions at the correct depth [19]. The ERC has indicated that mobile devices could play an important role in CPR outcomes [20,21]. However, it is important to note that further studies are needed to clarify exactly how these devices can provide effective guidance in real life-threatening situations [22].

Our review aims to examine smartphone applications and their potential to improve CPR quality, provide an overview of the smartphone technologies shaping this field, and examine their suitability. In addition, it aims to clarify the degree of their effectiveness and the ideal application areas, such as public places, homes, and sports events, where immediate CPR can significantly improve survival rates.

## Review

Methods

Although this article is a narrative review, we systematically searched for data to analyze the degree of effectiveness of its use in instructing CPR. The search was performed using PubMed and Cochrane academic databases. The last search was conducted on April 15, 2024. The keywords used were "GUIDED-CPR," AND/OR "SMARTPHONE APPLICATION," AND/OR "MOBILE APPLICATIONS," AND/"REAL-TIME FEEDBACK CPR," AND/PR "BYSTANDER CPR," AND/"LAYPERSON." The initial search yielded 154 articles. After excluding duplicate citations (33 articles), reviews, systematic reviews, meta-analyses, and pilot studies, 32 relevant articles were identified. Further review of titles and content led to the exclusion of 27 studies. In addition to the database search, two additional studies were identified manually through careful review of the reference lists of previously identified articles [23,24]. Finally, seven studies were added to the results section of this review. According to the final selection, two authors (L.V. and E.S.) selected the studies, and a third author (T.K.) helped resolve discrepancies if any, and made the final, consensual decision. All authors declare no conflict of interest regarding the final selection of studies.

Results

We included a total of seven studies in our final analysis, of which four were randomized controlled trials (RCTs) [19,25-27]. These studies involved random assignment of participants to different intervention groups, enabling comparisons of outcomes related to CPR performance and the effectiveness of smartphone applications or guidance methods. One study was a retrospective observational study analyzing existing data to observe outcomes related to CPR performance, with a particular focus on the effects of interventions in real-world scenarios [24]. One study was experimental [23], involving controlled experiments without randomization and focused on the effectiveness of specific interventions in a simulated environment. Finally, a feasibility study was included [28], which aimed to evaluate the feasibility and accuracy of a new chest compression depth (CCD) feedback algorithm implemented on a smartphone.

CPR quality was assessed using various outcomes listed in Table 1. The main indices include chest compression rate (typically 100-120 compressions per minute), chest compression depth (5-6 cm), chest compression percentage (>60%), compression depth at two minutes, and recoil (simultaneous with compression).

**Table 1:** Indicators of quality characteristics of chest compressions extracted from the smart applications CPR: cardiopulmonary resuscitation

Study	Indice	Description
Metelmann et al., 2021 [23]	No-flow time	Intervals during CPR in which there is no chest compression
Metelmann et al., 2021 [23]	Hand positioning	Correctness of hand placement when performing chest compressions
Plata et al., 2019 [19]	Compression depth	The recommended depth for each chest compression is 5–6 cm
Plata et al., 2019 [19]	Thorax release (per second)	Determine if, following compressions, the chest returns to its typical position
Plata et al., 2019 [19]	Compression rate at two minutes	The rate of chest compressions per minute, with a recommended rate of 100-120 compressions per minute
Song et al., 2015 [28]	Compression fraction	Percentage of time spent performing chest compressions (>60%)

Currently, numerous applications are available for free download by the users. Some of them are presented in Table 2. Most applications provide voice and picture instructions to BCPRs on performing high-quality chest compressions and proper actions. Additionally, the studies summarized in Table 2 approximate participants' performance in basic life support (BLS) implementation in real-world scenarios, primarily in manikins. Studies, aimed at demonstrating the benefits of applications, compare different groups or methods of guided CPR to determine the best possible combination.

**Table 2:** Studies on applications providing real-time CPR guidance CPR: cardiopulmonary resuscitation; PGC: PG-CPR; RMC: Rescue Me CPR!; U-group: uninstructed CPR; T-group: dispatcher-assisted telephone CPR; A-group: smartphone application feedback; TA-group: dispatcher-assisted telephone CPR + smartphone application feedback; CF: compression fraction

Study	Country	Applications	Groups (n)	Age (years)	Manikin (i); real-time (ii)	Outcome (based on the application used)
Marsh-Armstrong et al., 2023 [24]	USA	Me-CPR! (RMC)	CPR-certified (20); PGC app (20); RMC app (20)	>18	(i)	Positive effect: equivalent or superior to CPR performed by CPR-certified and superior to CPR-PG-CPR in almost every metric. No effect: time to first compression (CPR-certified: 34 ±10’’, PGC: 86 ±17’’, RMC: 55 ±6’’, p<0.01)
Metelmann et al., 2021 [23]	Germany	HELP NotFall	Control group (74); facultative group (64); mandatory group (60)	13-17	(i)	Positive effect: enhances the chest compression rate (p=0.004) and the compression depth (p<0.001). Negative effect: delays in the initiation of CPR (β=38.89 vs. 20.42, p<0.001)
Linderoth et al., 2021 [26]	Denmark	Live Video DA-CPR	Before video-instructed-DA-CPR (Ν=90); after video-instructed DA-CPR (N=90)	15-70	(ii)	Positive effect: significantly improved key CPR quality metrics (p<0.001). No effect: chest recoil (p=0.41)
Márquez-Hernández et al., 2020 [25]	Spain	Asistente-CPR	App group (66); telephone assistance group (62)	App group: 19.86 ±3.48; telephone assistance group: 21.15 ±6.61	(i)	Positive effect: checking the area is secure (p<0.001), opening of airways (p<0.001), emergency services contact (p<0.001), CF (p=0.03). No effect: skill level of CPR was equivalent to both groups (p=0.33)
Plata et al., 2019 [19]	Germany	Pocket-CPR	U-Group (25); T-group (25); A-group (25); TA-group (25)	U-Group: 31.2 ±13.5; T-group: 32.2 ±11.8; A-group:35.5 ±14.0; TA-group: 31.4 ±11.7 (p=0.551)	(i)	Positive effect: in terms of no-flow time (p<0.001), compression rate (p<0.001), proper hand placement (p=0.008 and p=0.009 between groups), thorax release (p<0.05 between all groups), time to first compression (p<0.001 and p=0.001 between groups). No effect: on the compression depth (p=0.051 and p=0.193 between groups)
Sarma et al., 2017 [23]	USA	CPR Assist Application	50 healthcare providers (58% ICU nurses)	>18	(i)	The application can precisely track compression depth (5-6 cm) and rate (100-120’)

According to the reviewed studies, CPR-guided smartphone apps are associated with certain limitations and a mix of benefits. In almost all metrics (chest compression fraction, compression rate, compression depth, high-quality CPR), for example, Mc-CPR! was found to perform better than or as well as CPR-certified individuals. The group using the application performed twice as well as the CPR-certified control group in high-quality CPR compared to the certified group (50% vs. 25%) in the set's compression cycles [26]. However, the application did not improve the time to first compression, where CPR-certified individuals had better performance (CPR-certified group: 34±10" vs. PGC app group: 86±17" vs. RMC app group: 55±6", p<0.001). This index is crucial because early chest compressions help maintain blood flow and supply oxygen to vital organs [9].

The HELP NotFall application also had a negative effect by delaying the start of CPR; the mandatory application use group experienced longer total hand-off time (time until first compression+time of all pauses during compressions) compared to the facultative group and the control group (p<0.001) [25]. On the other hand, this group's chest compression rate and depth significantly improved (mandatory group: 65.4 ±32.7 vs. facultative group 39.2 ±34.8 vs. control group 43.8 ±36.8, p=0.004 and 47.6 ±37.3 vs. 30.1 ±35.2 vs. 24.4 ±30.4, p<0.01 respectively). In addition, the Live Video DA-CPR app, the only tool tested in real-world OHCAs (p<0.001), significantly improved key CPR quality metrics like hand position and compression rate. This finding suggests that live visual feedback can effectively improve bystander CPR quality [25]. However, the same study failed to detect significant improvement in thoracic recoil before and after the initiation of the live video (p=0.41) [24]. Asistente-CPR and Pocket-CPR applications also demonstrated positive effects in checking for safety and proper hand placement on the chest, yet they did not significantly impact compression depth [19,27]. These results highlight the importance of improving these tools to cover all critical aspects of CPR performance comprehensively.

Discussion

Smartphone Applications That Provide Real-Time CPR Guidance

In recent years, many methods and devices have been employed to assist in CPR, such as dispatch-assisted CPR and CPR with instructions from a smartphone application [29]. Due to its positive impact on the quality of CPR, telephone CPR has been established in many centers in Europe and the USA [30-33]. However, due to the explanations required and the information requested, telephone instructions can significantly delay the start of the chest compressions, thereby reducing the overall outcome of a successful CPR application [34,35]. The use of smartphones has increased significantly in recent years, helping improve bystander CPR effectiveness through video-recorded instructions, voice instructions, metronomes, and compression depth measurement sensors [36-38].

To achieve a satisfactory result after an OHCA, it is crucial not only to perform CPR but also to perform high-quality chest compressions without wasting time. This quality is measured via smartphone applications while providing real-time CPR guidance with specific indices and has been validated in several studies [19,23,28]. These indices are presented in Table 1. Song et al. introduced a new feedback algorithm focusing on compression fraction, i.e., the proportion of time compressions performed during the overall time of CPR. This study concluded that smartphone applications may contribute to preserving a high compression fraction (satisfactory rate of ≥60%), which is necessary to keep blood flowing during cardiac arrest [28]. Another study found that app-supported groups achieved more accurate compression rates, aligning with the recommended guidelines of 100-120 compressions per minute [19]. In addition, it has been shown that real-time feedback significantly affects the no-flow time, the thorax release, the hand positioning, and the overall quality of CPR performance [23].

However, the limitations of the above studies should be taken into account as well. Age must be carefully considered when assessing CPR performance indices. The young age of BCPRs may not reflect the experience of the broader BCPR population, and older participants may need help to use this application properly [19,23]. In the study by Plata et al., it was suggested that participants over 60 years may have yet to be able to keep up the eight-minute CPR session [19]. Additionally, the duration of simulations in studies is shorter than in real-life emergencies. In the study by Metelmann et al. [23], the duration of chest compression in the simulation was two minutes, which may not be adequate to fully capture the required quality of CPR.

Moreover, CCD feedback-related limitations have been addressed. In the study by Song et al. [28], the CCD estimation algorithm could overestimate the chest compression depth if the patient was lying on a foam mattress because the algorithm also measures the compression of the mattress (not just the chest). Also, the algorithm used in the study was limited to fully detecting chest recoil with each compression. Full chest recoil is significant in CPR to allow the heart to refill with blood between compressions. The limitation is due to using an accelerometer, which calculates depth as the difference between the positive and negative peaks of compressions without considering whether full recoil is achieved.

Although the algorithm performed better than an existing application (PocketCPR) at some angle compressions, it still did not perform completely accurately at some other angle measurements (e.g., performing CPR on a pregnant woman or in an elevator). Plata et al. [19] found that smartphone application feedback could be contradictory at times, and this issue was exacerbated by the difficulty of maintaining the device stable during CPR (e.g., prompting "push faster" even when the correct compression rate was being achieved). Some practical suggestions to improve these limitations include using a backboard and integrating additional sensors to ensure effective monitoring of chest recoil or to manage oblique compressions [28].

Future Directions: Integrating Smartphone Applications into the Chain of Survival and Enhancing the Effectiveness of CPR Applications in the Community

A call for multidisciplinary action was issued recently by the "Lancet Commission to reduce the global burden of sudden cardiac death" [17]. The National Institutes of Health has emphasized the aspect of post-cardiac arrest and advanced resuscitation services. However, the first links in the survival chain - high-quality CPR, emergency response, and defibrillation - are also crucial for survival, highlighting the need for a balanced emphasis across all stages. Additionally, they recommended incorporating technological solutions for better resuscitation. Bystanders should use CPR feedback devices to receive accurate instructions on completing the steps of the BLS algorithm, as recommended by the American Heart Association [13,14,39].

Several studies evaluating the usefulness of these applications have demonstrated that they may significantly affect layperson performance (Table 1). Regarding CPR training, Baldi et al. [40] support incorporating real-time visual feedback into all BLS/AED courses to help laypersons achieve the training goals set by the ILCOR. Participants could perceive real-time visual feedback as a driving force behind improving their performance. An additional advantage of these devices is their ease of use; all that is required to operate them is a basic understanding of the included software, which any BLS/AED instructor can accomplish. However, the advantages mentioned in this study relate to training circumstances.

Regarding this concept, the QualiApp research project [41], a recent prospective-randomized simulation trial, assessed the quality of App-linked CPR feedback devices for guided CPR. They included four applications: the CorPatch Trainer, the CPRBAND AIO Training, the SimCPR ProTrainer, and the Relay Response. Their main finding was that the SIMCPR ProTrainer was the only technology that significantly improved CPR performance in chest compression depth compared to unassisted CPR (5.37±0.76, p<0.001). They also found that CorPatch Trainer (the only device with audio-visual instructions) brought better results on chest recoil rates vs. the other three devices (CorPatch Trainer: 49.00 ±42.20; p<0.01 vs. CPRBAND AIO Training: 37.03 ±39.90; p<0.01 vs. SimCPR Pro Trainer: 39.88 ±36.50; p=0.03 vs. Relay Response: 36.88 ±37.73; p=0.02). Also, chest compression depth (p=0.02), chest compression rate (p<0.001), percentage of correct chest compression depth/rate (p=0.03), and technical preparation time (p<0.001) all vary when performing CPR using the various feedback devices. Using feedback devices raised participants' confidence in performing CPR (p<0.001). However, the study again concluded that while some metrics show that laypeople's CPR performance improved with CPR feedback devices, none of the devices tested helped laypeople perform CPR adequately overall. More and better technical functionality is required to fully exploit the potential of CPR feedback devices and avoid a decline in CPR performance when laypeople use this technology.

In Greece, there is an application called "iSAVElives," which offers several other functions in addition to CPR guidance: it helps bystanders find the nearest AED using a national web map and sends a notification to every bystander using the app based on its distance [42]. However, application users must connect to a video platform to watch CPR guidelines (this can cause delays due to advertisements or internet connection delays), and the app's effectiveness in this field has yet to be standardized. Of the studies mentioned in Table 1, only one study from Denmark was conducted in real-time conditions [26]. Emergency dispatchers sent a text message with a secure link to bystanders' smartphones, enabling a live video stream from the scene. That allowed dispatchers to provide precise real-time guidance, such as correcting hand placement and ensuring proper compression depth and rate. Live video was used in 1.4% of emergency calls, with a high rate of success (82.2%). Using this guidance proved successful in improving the quality of CPR delivered by bystanders, potentially increasing the chances of survival during cardiac emergencies. However, the investigators noted several limitations, including the restricted use of video (due to the bystander's age, compliance, or the circumstances of the scene) and inconsistent verbal instruction.

**Figure 1:**
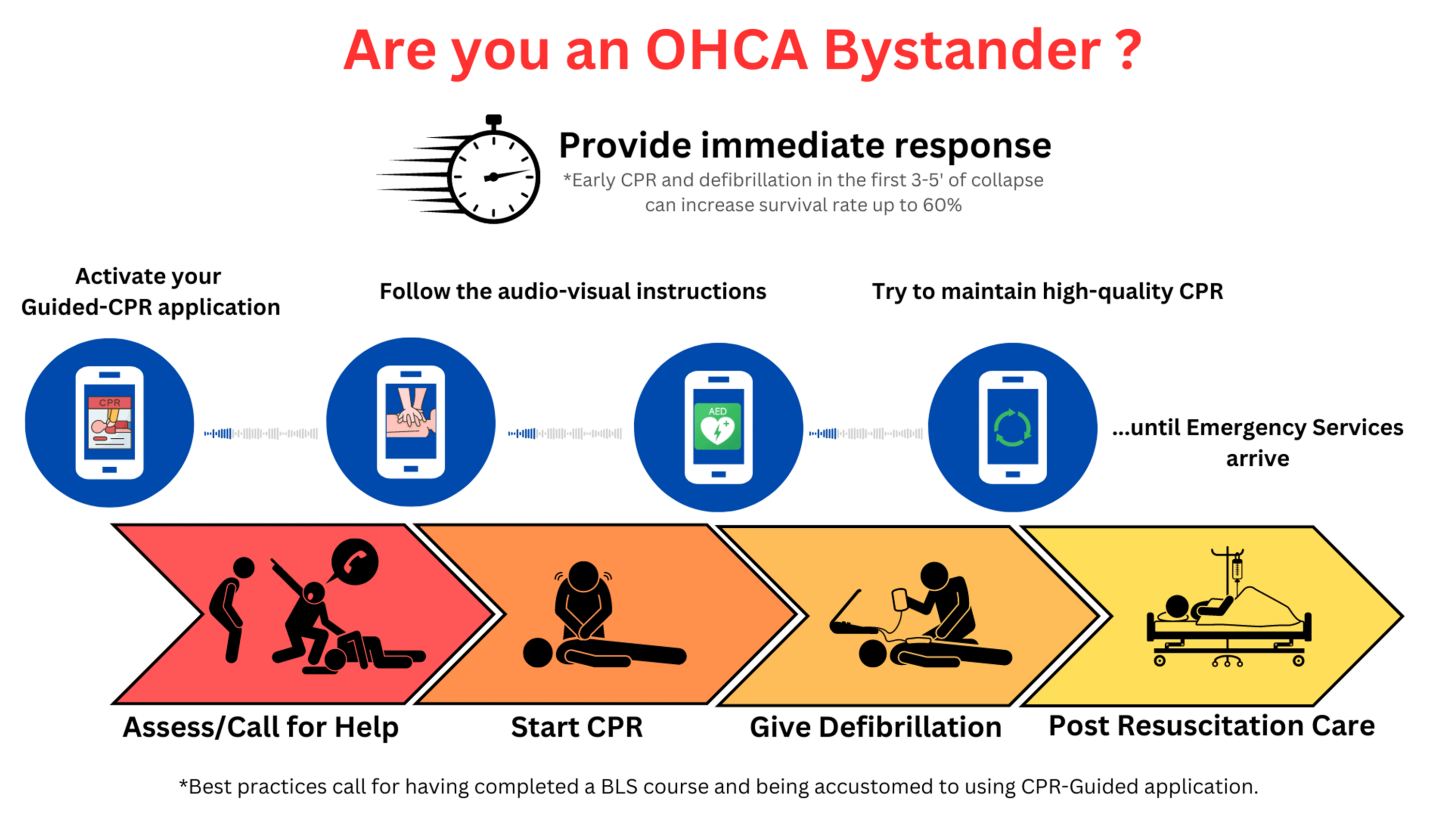
Guided CPR application incorporated into survival chain The information in the image comes from [14] and [21], and the overall message reflects the sum of the studies included in our review CPR: cardiopulmonary resuscitation; OHCA: out-of-hospital cardiac arrest

**Figure 2:**
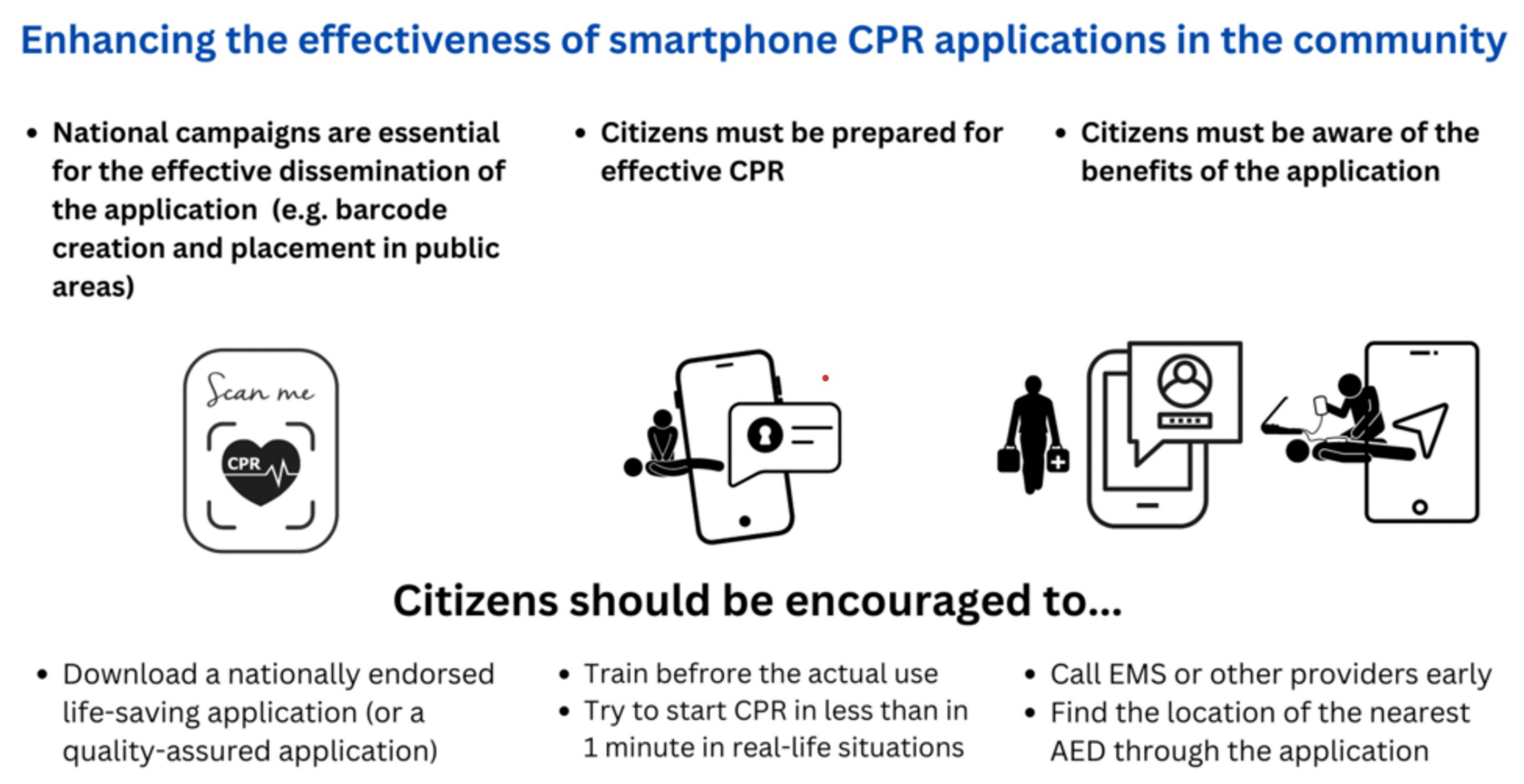
Guided CPR application in the community This image was created by authors based on official sources. The information in the image comes from references [14], [17], and [21], and the overall essence reflects the sum of the studies included in our review CPR: cardiopulmonary resuscitation; EMS: emergency medical services; AED: automated external defibrillator

Based on our research, we recommend that laypersons become familiar with these applications during BLS training or through CPR campaigns. Renewing the chain of survival could also provide additional benefits for spreading knowledge about guided CPR (Figure 1). Additionally, standardized applications tailored to each country's specific needs and guidelines should be freely released to the public (Figure 2). This initiation would ensure a more precise evaluation of Guided CPR quality outcomes across different regions. Also, the creation of a standardized application can help individuals confidently assist in emergencies, knowing that guidance and necessary resources are just a 'click' away. Mobile technology can be optimized by developing applications with simplified, clear steps that can be voice-activated during an incident, thereby reducing the time without chest compressions (to start CPR in less than one minute after the victim collapses).

Following standardization, the next step is to familiarize the public with these CPR applications (Figure 2). Public service announcements regarding local applications, citizen familiarization with these programs for potential bystander practitioners, and awareness-building regarding the new chain of survival are all necessary. We recommend publishing public notices detailing their use and benefits (programs in schools, public workplaces, community centers, etc.) or placing posters with barcodes for downloading applications in public areas (public transport stops, schools, gyms, community centers). Educational campaigns would be required to raise awareness about integrating smartphone applications as a crucial link in the chain of survival (Figure 1). With the help of emergency protocols and national health guidelines, this could be achieved more successfully. In addition, a support infrastructure must be created so that EMS can assist BCPRs using these applications in emergencies. Adopting this approach can improve the utility of guided CPR, benefiting both emergency professionals and the general public in the efficient use of this technology. Another advantage would be more accurate documentation of the response rate of BCPRs and the incidence of OHCAs.

To summarize this section, guided CPR through smartphone application should be designed to empower community engagement and foster a culture of empathy and preparedness. Attention and action by government authorities, focusing on the first links in the chain of survival, are crucial in this matter.

Limitations

The following significant limitations of the study should be noted when evaluating its findings on the usefulness of smartphone applications for CPR guidance. Firstly, most research done in controlled settings, such as that with manikins, may not accurately represent real-world circumstances. Participants may act or perform differently in real-world emergencies. Additionally, various studies frequently employ various smartphone applications, each with unique features and feedback systems. Because of this variability, it may be challenging to directly compare results and identify the application features that significantly improve CPR outcomes. Another problem is the accuracy of the technology in providing feedback, such as sensors and algorithms. Inaccuracies in these technologies may result in incorrect guidance.

Additionally, the included studies may have been biased in several ways. Although most RCTs used randomization, some studies may have had performance and detection bias due to a lack of blinding between participants and assessors. The fact that participants were aware of the intervention they received and how it might have affected their performance is one of the main causes. Furthermore, because the design of retrospective observational studies is non-randomized, there is a greater chance of selection and detection bias. Due to these possible drawbacks, it is necessary to evaluate the findings of chosen studies cautiously. Furthermore, it is only sometimes evident how much users are willing to use these applications in emergencies. Essential elements that can significantly impact how effective these applications are user engagement and compliance. Finally, smartphone applications' usability and content may not have undergone adequate quality control, which could result in non-compliance with updated guidelines.

## Conclusions

Smartphone applications in CPR offer significant potential to improve emergency response, particularly among laypersons. Their easy accessibility, real-time guidance, and the ability to empower non-medical personnel to perform life-saving techniques until professional help arrives are undeniable. However, we must also acknowledge the limitations, such as the potential delay in initiating CPR due to the time required to activate and navigate these apps. To optimize their routine use, we need to urgently conduct more extensive, multicenter RCTs in real cardiac arrest scenarios. Developing standardized applications tailored to each country's specific guidelines can ensure consistent quality and measurement of CPR. Therefore, it is crucial to integrate these technologies into BLS training programs to ensure users can use them effectively in emergencies. The future of lay CPR implementation looks promising, as we combine technology and education to make life-saving interventions accessible to all.
